# Influencing factors of the treatment level of elderly care workers and their career development prospects

**DOI:** 10.1186/s12877-023-04084-w

**Published:** 2023-06-09

**Authors:** Kaichang Cui, Fei Yang, Ruihan Qian, Chenmei Li, Mengting Fan

**Affiliations:** 1 Social Security Research Center, Shanghai University of Engineering Science, Shanghai, China; 2 School of Management, Shanghai University of Engineering Science, Shanghai, China

**Keywords:** Elderly care workers, Treatment level, Influencing factors, Career development prospects

## Abstract

**Background:**

The increasingly aging population in current China has encouraged the emergence of the diversified and multi-level elderly care service industry, and the demand for high-quality elderly life with the help of elderly caregivers continues to grow.

**Methods:**

Based on the existing questionnaire data, this article explores the influencing factors of the treatment level of care staff, and explores their future development prospects.

**Results:**

The results show that whether they have participated in relevant vocational skills competitions, whether they have worked overtime, whether they have overtime wages, and their monthly income have significant effects on their satisfaction of treatment levels. Elderly care workers who have participated in skills competitions are more satisfied about their salary. In addition, workers who rarely and occasionally work overtime are more satisfied compared with those who have never worked overtime; Caregivers with a monthly income of 5,000–6,999 yuan are more satisfied with their salary and treatment than those with below 3,000 yuan.

**Conclusion:**

Therefore, in order to better match the supply and demand of care workers, we should provide formal training and skill competitions for them, appropriately increase their salary level and reasonably arrange their working hours, so as to attract more professional talents into elderly care industry.

**Supplementary Information:**

The online version contains supplementary material available at 10.1186/s12877-023-04084-w.

## Background

With the increased number and proportion of the aging population in almost every country over the globe, it is fair to conclude that the whole world is facing an unprecedented era of aging. Almost all sectors of society are affected by population aging trends, including labor and financial markets, demand for goods and services such as housing, transportation and social security, and even family structure and intergenerational relationships. Meanwhile, the demand for institutional care service and long-term care workers is on the sharp rise, as the number of elderly people and the proportion of the elderly with dementia and disability continues to increase.

Some western countries have their own experiences in the development of the profession of nursing staff. Germany likewise has a gigantic demand for elderly care workers, and the demand for elderly care workers is also comparatively large. Data show that 0.12% of German citizens are engaged in pension work, and the number has surpassed 1 million. Among them, 68.2% of nursing staff work in elderly care institutions, and the rest are home-based elderly care services. ​Consequently, Germany has given great support to the nursing staff in terms of skills training, financial security, and institutional norms. The United States, Italy, Spain and other countries have accumulated rich experience in the construction of aging nursing staff, including the establishment of a comparatively complete hierarchical nursing mechanism and a reasonable quantitative form to standardize nursing staff. In Japan, there are two main types of elderly care facilities: Outpatient Rehabilitation (OR) and Outpatient Day Long-Term Care (ODLC). The former is equipped with more skilled aging service personnel, who can provide a series of high-quality services for the elderly, including daily life care and disease rehabilitation. For seniors who require long-term care, there is no difference between the two services. ​Nevertheless, compared to ODLC services, OR services play an increasingly important role in preventing health deterioration [1]. On the other hand, the aging trend of developing countries such as my country is expanding, resulting in a severe shortage of aged service personnel, which has become a major obstacle to the development of the elderly service system. Data show that in 2021, there will be about 75,000 professional nursing staff in Shanghai nursing institutions, accounting for 1.29% of the total aged population in Shanghai. There is a shortage of elderly care workers, and there is a severe imbalance between supply and demand of elderly care services.

Population and labor resources are common late-mover advantages in developing countries. Faced with the huge demand for long-term care employees, how to enhance their satisfaction with wage and treatment, effectively evolve the long-term care team, and improve the employment quality of the long-term care industry has become an urgent problem to be resolved. solutions in developing countries.

## Review

Researchers have conducted in-depth studies on the salary and performance bonus, mental health and job satisfaction of care workers. In terms of their salaries, that there are obvious inequality in payment for medical staff of different races and genders, thus effectively increasing the salary of nursing staff in disadvantaged positions can reduce the poverty rate to a certain extent. Therefore, they argue that significant changes to the compensation structure in care service industry are needed [2].In terms of the influencing factors of job satisfaction of elderly caregivers, Hasson H et al. believe that the two factors of job burnout and career development have a significant relation with the satisfaction of elderly caregivers, so future intervention measures should emphasize on counteracting work-related exhaustion and improving caregiver competency to improve their job satisfaction [3]. In terms of nurses' welfare and patients' satisfaction, nurses' dissatisfaction with their jobs can lead to costly labor disputes, staff attrition and patient risk. What's more, in hospitals where nurses are more dissatisfied about their treatment, patient satisfaction degree is also lower. Therefore, it is advocated to improve the working conditions of nurses, thereby improving the satisfaction of both nurses and patients and the quality of care service [4].

In terms of the mental health of care workers, the influence of gender should be studied to clarify the relationship between the psychological stress and job burnout faced by nursing staff in the work process, and to find solutions to their psychological problems [5]. Scholars such as Kirsten Moore attempt to conduct a cross-sectional analysis of elderly care workers through the Geriatric Depression Scale, demographics, personality characteristics, attitudes toward aging and other characteristics of caregivers to identify the factors relevant with depression of elderly care workers. The results find that long working hours and high neurotic level have a significant impact on the depression of elderly care workers, so countermeasures can be carried out accordingly to solve this problem [6].

In terms of the relationship between job burnout and productivity of nursing staff, there is a significant negative correlation between job burnout and productivity, while there is a significant positive correlation between personal achievement and productivity, so they suggest reducing the workload of nursing staff to improve the quality of patient care and better their work experience [7]. Regarding the relationship between emotions and job burnout and turnover, the death of the elderly in the care service institution will have a significant impact on nursing staff, and the sadness caused by the death will lead to job burnout, which will result in the rise of turnover rate. Therefore, strengthening mental health support for nursing staff is essential [8].

In terms of improving the job satisfaction of elderly care workers, improve the working conditions of elderly care workers, i.e. appropriately increase their salary, improve the arrangement of personnel, and enhance the service capacity of long-term care institutions to improve their job satisfaction [9]. In terms of talent team construction, the overall quality and treatment of elderly care workers can be improved by increasing their sense of honor, providing work incentives, paying attention to mental health and improving team building [10]. In terms of improving the treatment level of elderly care workers, their current treatment is at a relatively low level and can be improved from the perspective of government, i.e. ensuring the equity of welfare facilities provided by the central government, strengthening publicity to increase social respect for care workers, improving the support from local government for the treatment of long-term care workers, and increasing the accountability of local governments to improve the treatment of long-term care workers [11]. In terms of the factors affecting the salary of nursing staff, compared with other jobs, the salary of nursing staff is low and the labor conditions are poor. Meanwhile, they find that the location of care service institutions will affect the salary of care workers. Therefore, they suggest to strengthen public nursing institutions and establish private service companies [12]. As for the attractiveness of salaries, working hours and benefits to family caregivers, in order to better attract family caregivers, home care institutions should take measures such as raising wages and adjusting working hours [13]. In terms of the relative compensation of nursing work, nursing work will bring wage losses, and therefore collective action is needed to ensure the support of the government and other stakeholders for nursing work [14]. With regard to the wage and income inequality of nursing, income inequality and low income level has become the characteristics of the nursing industry [15]. In addition, the participation of nursing staff can improve the existing nursing structure and the continuity of the structure in the hospital [16].

In terms of the influencing factors of nurses' salary, the standard salary level of nurses mainly depends on nursing experience and professional knowledge [17]. Based on the data collected, a subset of nurses felt they were being paid a salary that did not match their work experience, in contrast to those in the healthcare industry being paid more [18]. the geographic location of nurses' jobs and the size of the institution also had a significant impact on their salary levels [19]. Thrall and Hudson conducted a salary survey of head nurses and found that the salary level of head nurses was affected by working years, and when the working years exceeded ten years, their wages generally reached a higher level [20]. The hospital scale, fund characteristics and nursing service system jointly affect the salary level of nurses [21]. Gender differences in nurse practitioner salaries, controlling for work environment and demographic factors, and found that for newly graduated nurse practitioners, there was a gender difference in salary that widened over time, and present in all clinical specialties [22]. Male nurse practitioners have a certain gap compared to female nurse practitioners in terms of specialties, facilities and positions, and the most important thing is that the pay gap between the two has not narrowed over time [23].

Existing studies have explored the aspects of nursing staff's salary, mental health, job satisfaction, etc., especially discussing the influencing factors of nursing staff's salary, nursing experience, working years, work organization and gender, etc. All factors can have a large impact on the salary of caregivers, but the existing research is usually based on the research of the staff of the whole nursing industry, and the aged care staff, as an integral part of the nursing industry, should carry out special and detailed research. Most of the existing researches discuss the influencing factors of compensation from a macro perspective. This paper studies the factors affecting the treatment level of elderly care workers from a micro perspective. Based on the above content, this paper focuses on analyzing the factors affecting the treatment of elderly nursing staff, in order to solve the current imbalance between supply and demand of nursing staff, and explore the future career prospects of nursing staff.

## Methods

### Data sources

Cross-sectional data is used in this study from the survey on the elderly care workers in Area B of Shanghai. The survey first selected 40 elderly care institutions, 30 elderly day care centers, 27 nursing homes/stations, 10 community elderly care service organizations and 8 community comprehensive elderly service centers in the whole district. Then, based on the method of Stratified Sampling, the final subjects were selected among the above organizations, namely 8 elderly care institutions, 6 elderly day care centers, 3 nursing homes/stations, 2 community elderly care service organizations, and 2 community comprehensive elderly service centers. The number of questionnaires conducted in the institutions was distributed according to the number of beds for elderly (10 questionnaires for institutions with < 200 beds, and one questionnaire per 20 beds for institutions with > 200 beds) and the maximum number of questionnaires per institution is 30. In the end, a total of 366 questionnaires were received and 363 was valid, with the effective rate of 99.2%.

### Data collection

The treatment level of care workers is determined by the method of self-evaluation by answering the question "Are you satisfied with your treatment level" in the questionnaire as the measurement standard. Since the option "neither satisfied nor dissatisfied" does not reflect the general inclination of satisfaction, we assigned the value of 2 to both "very dissatisfied" and "somewhat dissatisfied", and assigned the value of 1 to both "relatively satisfied" and "very satisfied".

As for the influential factors in determining the treatment level, this study selected the participation of elderly care workers in vocational skills competition, income level, skills training and overtime work as explanatory variables. Specifically, the answers of the respondents in the questionnaire are used as the measurement standard. Regarding vocational skills competitions, respondents need to answer "Have you participated in relevant vocational skills competitions" and we assign the value of 2 to "Yes", and 1 to "No". The income level is measured by answering "What is your current income?" and similarly we classified the answer options into 4: "Below 3000" is assigned 1, "3000–4999" is assigned 2, "5000–6999" is assigned 3, and "7000 and above" is assigned 4. The income classification criteria used in the original questionnaire in this paper are below 1000 yuan, 1000 yuan to 2000 yuan, 2000 yuan to 3000 yuan, 3000 yuan to 4000 yuan, 4000 yuan to 5000 yuan, 5000–6000 yuan, 6000 yuan to 7000 yuan, and 7000 yuan to 8000 yuan More than 8,000 yuan. However, in the actual collection process of the questionnaire, we could not guarantee that the number of interviewees within each income range was close, and some interviewees with income levels might be 0. Therefore, after clearing and screening the questionnaire results, we re-classified the income levels of interviewees. The division is mainly based on the income level of Shanghai. According to the Shanghai Municipal Human Resources and Social Security Bureau, the city has adjusted the minimum wage from July 1, 2021. The monthly minimum wage standard was adjusted from 2,480 yuan to 2,590 yuan, and the Municipal Human and Social Affairs Bureau, the Civil Affairs Bureau and the Municipal Health Commission jointly issued the "Guidance on the Establishment of Salary rating System for Elderly Care Workers", which clearly put forward the standard and specific amount of salary rating. The salary rating of elderly care workers is determined according to their skill level and years of service for the elderly. There are 6 levels and 23 levels. The basic salary standard for the salary grade of old-age care workers is tentatively set at 2,600 yuan/month. Therefore, after comprehensive consideration of relevant information, this paper sets the first stage of the salary level of old-age care workers as below 3,000 yuan. Of the 23 pay grades, the median salary is 4,810 yuan, so the second tier is divided into 3,000 to 4,999 yuan. The third grade is divided based on the fact that since July 1, 2021, the upper and lower limits of Shanghai's social security base in 2021 will be 5975–31,014 yuan. In consideration of the actual income of elderly care workers, this paper selects 5000–6999 yuan as the third grade of classified income. Finally, the above "Guidance on the establishment of salary rating system for elderly care workers" shows that the highest income level of elderly care workers can reach more than 7000 yuan, so 7000 yuan or above is taken as the last level of income division. In order to explore whether the professional skill level of care workers will affect the treatment level evaluated by themselves, we use "Have you participated in the training of elderly care skills" to measure their skill levels, and the value of 2 is assigned to "Yes" and 1 to "No". For overtime work, in order to know whether the frequency of overtime work will influence the treatment level evaluated by themselves, we use "Have you been required to work overtime" and assign the value of 1 to "Never", 2 to "Rarely", 3 to "Occasionally", 4 to "Sometimes", and 5 to "Often".

### Statistical breakdowns

The data are expressed in terms of frequency, weighted ratio, and mean ± difference. Based on the analysis of the basic information of care workers in the previous part, binary logistic regression analysis was carried out to explore the influencing factors of the treatment of care workers in Shanghai. Since there are two answer options "Yes" and "No" for the dependent variable "Are you satisfied with your treatment", a binary logistic regression model is selected to ensure effective analysis of the influence of independent variables on dependent variables given the same other variables. The model for binary logistic regression analysis on the dependent variables is as follows:

According to the definition of variables in this research, the satisfaction of the care workers with the treatment level is defined as the event occurrence, and a binary logistic regression model is used to analyze the impact mechanism of all factors on their feelings (satisfaction or dissatisfaction) of the treatment level. All statistical analysis were conducted using IBM SPSS software v23.0 for Windows.

## Results

### Basic information of elderly care workers

The age of the respondents is mainly between 47 and 57 years old, with 49 on average; the elderly care service institutions where they work are mainly established and operated privately(private non-enterprise units) or by public sectors. Among the elderly care workers surveyed, the proportion of married women is higher; 77.9% of the workers are of junior-high-school education and below. The income of most workers is between 3000–4999 yuan, accounting for 73.6%; among the respondents, 196 (53.9%) of them are rural migrants from other provinces, and only 31.6% have Shanghai *hukou*. Therefore, nearly half of the workers live in the care service institutions, and 252 (69.4%) of the workers engaged in agricultural work or in the service industry, etc. before working in the current care service institution. As shown in Table 1.

**Table 1:** Analysis of the basic information of the respondents

Basic information		Number (%)	Basic information		Number (%)
Gender	male	44 (12.1)	Education level	≤ primary school	89 (24.5)
	female	319 (87.9)		Junior high school	194 (53.4)
Salary	< 3000 yuan	55 (15.2)		High school	47 (12.9)
	3000–4999 yuan	267 (73.6)		Technical secondary school	22 (6.1)
	5000–6999 yuan	30 (8.3)		Junior college	6 (1.7)
	> 7000 yuan	11 (3.0)		≥ Bachelor	5 (1.4)
Marriage	married	332 (91.4)	*Hukou* category	Shanghai urban	63 (17.3)
	widowed	18 (4.9)		Shanghai rural	52 (14.3)
	devoiced	8 (2.2)		Non-Shanghai rural	196 (53.9)
	unmarried	5 (1.3)		Non-Shanghai urban	52 (14.3)
Offspring	0	35 (9.6)	Institution category	Established by public sectors, run privately;	38 (10.4)
	1	216 (59.5)		Established and run privately (private non-enterprise units);	161 (44.3)
	2	91 (25.1)		Established and run by the public sector (private non-enterprise units);	13 (3.5)
	≥ 3	21 (5.8)		Public institution	10 (2.7)
Residential arrangement	With spouses	81 (22.3)		Established and run by the public sector;	90 (24.7)
	With spouses and children	94 (25.9)		Established and run privately	10 (2.7)
	With children	12 (3.3)		Established and run privately(for profit)	10 (2.7)
	Along	24 (6.6)		Established by public sectors, run privately(private non-enterprise units)	22 (6.0)
	In the institution	152 (41.9)			

### Elderly care service and supply

The analysis of the service supply of elderly care workers found that although many elderly care workers had been engaged in this work for over 5 years, half of the workers had no work experience as elderly care workers before; On-the-job training is provided for them in most elderly care service institutions. More than 95% of care workers are willing to participate in nursing skills training for the elderly, and 348 (95.8%) have participated in such trainings. 254 (70.0%) of them have acquired the professional qualification certificate for elderly care, but most of their certificates are at junior level. More than half of the respondents do not have the qualifications for services included in the Long-term Care Insurance, nor have they participated in relevant vocational skills competitions. Care workers mainly provide institutional services for the elderly, mainly living care, and they are responsible for nursing 6–10 elderly people per day on average. Most of them work more than 8 h a day but rarely work overtime, and overtime wages will be paid regularly; the respondents believe that they lack the most theoretical knowledge and innovative service in terms of professional competence. They are mainly contracted workers outside the authorized personnel quota, and only a small number of them have entitled the five social insurances and the housing fund; 316 (87.0%) are satisfied with the current salary and treatment.

There will be volunteers participating in elderly care activities in daily work, which are well-received by the elderly; 274 (75.4%) of the elderly in the survey have a supportive attitude towards elderly care institutions, and 310 (85.3%) of them are relatively satisfied with the service quality; 83.1% elderly care workers believe that this occupation is secured with a promising career prospect. 271 (74.6%) of them will continue this job, but the psychological stress and work intensity of this job are part of the barriers for them to perform nursing work in the long run. As shown in Table 2.

**Table 2:** Analysis of the service and supply of elderly care workers

Service supply		Number (%)	Services supply		Number (%)
Previous experience as care workers	No	198 (54.5)	Participated in training	No	15 (4.2)
	Yes	165 (45.5)		Yes	348 (95.8)
How long being engaged in aged care industry	0–1 year	36 (9.9)	Competence they lack the most	Theoretical study	132 (36.4)
	1–2 year	52 (14.3)		Nursing practice	72 (19.8)
	2–3 year	49 (13.5)		Innovative service	122 (33.6)
	3–5 year	56 (15.4)		Work engagement	37 (10.2)
	5–8 year	73 (20.1)	Qualifications for services included in the Long-term Care Insurance	No	182 (50.1)
	8–10 year	40 (11.0)		Yes	181 (49.9)
	> 10 year	57 (15.7)	On-the-job training	No	6 (1.6)
Professional qualification certificate	No	109 (30.0)		Yes, irregularly	79 (21.7)
	Yes	254 (70.0)		Yes, regularly	278 (76.5)
Level of the certificate	Junior(level 5)	175 (48.2)	Participated in skill competition	No	226 (62.3)
	Middle(level 4)	58 (16.0)		Yes	137 (37.7)
	High(level 3)	21 (5.8)	Service provided for the elderly	Living care	333 (91.7)
Average day-off per month	1 day	27 (7.4)		Technical care	123 (33.8)
	2 day	23 (6.3)		Rehabilitation care	96 (26.4)
	3 day	11 (3.0)		Psychological care	171 (47.1)
	4 day	110 (30.3)		Training guidance	22 (6.0)
	5 day	10 (2.8)		Care management	62 (17.0)
	6 day	24 (6.6)		others	24 (6.6)
	others	158 (43.5)	Daily working hours	3–5 h	1 (0.3)
Average number of elderly cared for each day	≤ 5	66 (18.2)		5–8 h	56 (15.4)
	6–10	220 (60.6)		8–10 h	137 (37.7)
	11–15	43 (11.8)		> 10	169 (46.6)
	≥ 16	34 (9.4)	Work overtime	Never	28 (7.7)
Self-evaluation of salary	dissatisfied	47 (13.0)		rarely	119 (32.8)
	satisfied	316 (87.0)		occasionally	111 (30.6)
Problems in care work	Low wages and long working hours	91 (25.0)		sometimes	42 (11.6)
	Difficulty communicating with the elderly	125 (34.4)		Often	63 (17.4)
	Not respected by the public	139 (38.2)	Overtime wage	No	58 (16.0)
	Not respected by the elderly and their families	92 (25.3)		Yes, paid irregularly	15 (4.1)
	Poor welfare	33 (9.0)		Yes, paid regularly	290 (79.9)
	No pay-rise for a long time	31 (8.5)	Social insurance covered by the organization	Pension	199 (54.8)
	Non-local employment difficulty	48 (13.2)		Medical insurance	171 (47.1)
	Others	56 (15.0)		Unemployment insurance	125 (34.4)
Career prospects	Very pessimistic with problems in salary and skills acquisition;	31 (8.5)		Injury insurance	125 (34.4)
	Limited development with occupational restrictions;	23 (6.3)		Maternity Insurance	324 (89.3)
	Neutral attitude, very stable;	181 (49.8)		Accident insurance	81 (22.3)
	Very optimistic with huge improvement potentials	121 (33.3)		Housing fund	139 (38.2)
	Others	7 (1.9)		Others	204 (56.0)
Job hopping	No	84 (23.1)	Barriers in work	Low salary	91 (25.0)
	Very unlikely	53 (14.6)		high work intensity	140 (38.5)
	Neutral	134 (36.9)		Psychological stress	173 (46.7)
	Very likely	50 (13.7)		Nursing stress	52 (14.3)
	Yes	42 (11.5)		Demanding nursing skills	69 (19.0)
				Bad relations between co-workers	4 (1.1)
				Bad working environment	3 (0.8)
				Others	45 (12.3)

### Analysis of the influencing factors of treatment satisfaction of elderly care workers

In order to further explore and analyze the influencing factors of treatment satisfaction of care workers, binary logistic regression analysis was carried out, and salary satisfaction was used as the dependent variable. After transforming all categorical variables into dummy variables, they were used in the binary logistic regression equation, and the stepwise regression method was adopted. The entry level was α = 0.05, and the exclusion level was β = 0.10.

According to the results of the Hosmer and Lemeshow Test shown in Table 3, it is found that the significance of the constructed model was greater than 0.05, indicating that there is no significant difference between the predicted and true values of the model, and the model is considered to have a good fit. In addition, the results in the classification table in Table 4 found that the prediction results of the model are correct at 87.6%, and the model is considered to have a high reliability.

**Table 3:** Hosmer and Lemeshow Test

Chi-square	df	Sig
4.939	7	.667

**Table 4:** Classification table

Observed		Predicted		
		Self-evaluation of salary		Percentage Correct
		1	2	
Self-evaluation of salary	1	6	41	12.8
	2	4	312	98.7
Overall Percentage				87.6

According to the binary logistic regression analysis results shown in Table 5, it is found that whether the respondents have participated in vocational skills competitions, whether they have worked overtime, whether they are paid with overtime wages, and their salary have a significant impact on their satisfaction of treatment. Participation in vocational skills competitions has a positive impact on their salary satisfaction (OR = 2.176, 95%CI: 1.025–4.621); In addition, "rare and occasional overtime work" has a positive impact on their satisfaction (OR = 4.847/6.368, 95%CI: 1.480/1.212–15.878/33.458), compared with "never working overtime"; and the respondent who have received regular overtime wages are more satisfied with their own salary level than those who have not (OR = 4.661, 95%CI: 2.047–10.611). Regarding their income, those whose salary is between 5000–6999 yuan are more satisfied with their treatment than those with 3000 yuan (OR = 10.320, 95%CI: 1.150–92.598).

**Table 5:** Binary logistic regression analysis results of factors influencing the service supply of care workers

Variables	B	S.E	Sig	Exp(B)	95% C.I.for EXP(B)	
					Lower	Upper
Constant	-1.632	.846	.054	.195		
Participated in skill competition						
No						
Yes	.778	.384	.043	2.176	1.025	4.621
Work overtime						
Never						
Rarely	1.578	.605	.009	4.847	1.480	15.878
Occasionally	1.851	.846	.029	6.368	1.212	33.458
Overtime wage						
No wage						
Yes and paid regularly	1.539	.420	.000	4.661	2.047	10.611
Salary						
≤ 3000 yuan						
5000–6999 yuan	2.334	1.119	.037	10.320	1.150	92.598

## Discussion

This study mainly focuses on the satisfaction of the salary and treatment of care workers, in order to understand the factors that affect their treatment level, explore their career path, and predict their development in the near future. The results of this study show the following basic characteristics of the care workers in Shanghai: the average age is 49 years old; most of them are married women with one child, with the education level of junior high school and below; most of them are rural migrants from other provinces, living with their children or living in the work place; most of them engaged in rural work or service industry before working as caregivers; most of the respondents have participated in nursing skills training for the elderly, and 70% of the respondents have professional qualification certificates. They are basically satisfied with the current salary and treatment, and optimistic about the job prospects because of the sense of stability. It is also found that whether they have participated in relevant vocational skills competitions, whether they have worked overtime, whether they have overtime wages, and their salary have a significant impact on their satisfaction of treatment. Based on the Social Support Theory, there are both formal and informal social support for care workers [24], and more formal and informal social supports are both conducive to maintaining their occupational stability.

This result has a certain similarity with the research results of Nancy, Paula, Park Renya and others. They found that the current salary level of elderly care workers is at a relatively low level, and there is inequality in the level of treatment. Collaborative approach to raise the current level of satisfaction of elderly care workers with salary packages. With the gradual deepening of the aging degree, the problem of old-age care has become an important problem to be solved urgently in our country. The development of the elderly care worker industry has greatly eased residents' anxiety about elderly care. At present, there is a serious shortage of supply in the elderly care worker industry in our country, the main reason of the problem is the low level of wages. Therefore, this article dives into the factors affecting the treatment level of elderly care workers, explains and analyzes the current salary satisfaction problem of nursing staff from the three aspects of occupational level, work intensity and salary level, and provides solutions to alleviate the problem, so as to alleviate the problem of the unbalanced supply and demand, and at the same time promote the healthy and sustainable development of the elderly care worker industry.

According to the research results, the main conclusions and suggestions for measures to solve the problem of the unbalanced supply and demand of elderly care workforce are summarized in the following.

First, the high work stress can easily induce mental health problems. Elderly care workers mainly provide institutional services for the elderly, i.e. living care, and 41.3% of the workers live in elderly care institutions, which means their daily work and life are blended together, and they usually work over 8 h per day. Although it is not regarded as "overtime work", the overall working time is quite longer, compared with that of other occupations. The research has found that those who rarely or occasionally work overtime are more positive about payment compared with workers who never work overtime. The possible reason is that overtime work can increase the income of employees since the institution will regularly pay them overtime wages, so care workers will not feel reluctant about it. In addition, due to the large workload and the fact that they are “not respected by the general public (38.2%)” and “not respected by the elderly and their families (25.3%)”, this kind of social discrimination is likely to negatively influence their mental health [25]. The intensive psychological stress has led to the loss of professionals to a certain degree, which is detrimental to the long-term development of this industry. Therefore, elderly care service institutions should reasonably plan the working hours of elderly care workers such as implementing a shift system to ensure that care workers have adequate rest while meeting the care needs of the elderly, or making compensation by improving their benefits. In addition, institutions should pay attention to the mental health of care workers and help them get rid of negative feelings in a timely manner, such as organizing cultural and recreational team building activities monthly to better their work experience.

Second, the monthly income level of these workers is largely affected by their professional competence. The data shows that most of the elderly care workers have participated in on-the-job training provided by institutions, such as how to perform living care and psychological care, but they are not trained for medical care skills. Only 25.9% of the respondents are able to provide medical care services. For elderly people with special needs, such as the advanced ages lacking self-care ability, care workers need to acquire relevant medical care skills correspondingly. However, restricted by their education level and declining cognitive ability at old ages, these care workers are in disadvantage of acquiring new nursing knowledge, eventually leading to a general lack of professional competence, failing to meet the demands of the elderly for a long time [26]. Meanwhile, this study found that half of the employees were engaged in rural work or service industry with no previous nursing experience before working as elderly care workers. The undiscriminating personnel recruiting process in this industry naturally leads to a low income level of elderly care workers. Therefore, it is suggested that elderly care service institutions should conduct pre-job training for applicants, actively organize regular on-the-job training for them, invite professional nurses to share medical and nursing knowledge to them, and introduce talents from professional nursing colleges. In this way, we can invigorate the whole industry with younger practitioners and improve the overall quality of employees. In addition, the civil affairs department should actively organize vocational skills competitions for care workers to motivate employees for self-improvement, strengthen their professional ability and promote the high-quality development of elderly care services. Meanwhile, elderly care service institutions and relevant government departments should clarify incentives and benefits, ensure the income level of care workers are consistent with their professional abilities, and increase their treatment level in an appropriate way.

## Conclusions

Based on the survey among elderly care workers in Shanghai, this research aims to analyze and forecast the development prospects of elderly care by analyzing the collected data. It is found that the salary, participation in vocational skill competitions, overtime work and overtime wage payment have a great impact on care workers' satisfaction of treatment. Meanwhile, the data shows that currently most of the workers engaged in the nursing care industry are women with advanced age and low education. Graduates from vocational and technical colleges majoring in the elderly care are often reluctant to work as elderly caregivers or involve in the elderly care industry. Salary satisfaction has a great impact on people's work engagement. Therefore, in order to improve the enthusiasm of care workers and attract more young talents into the elderly care industry, it is suggested that relevant government departments should increase the monthly income of care workers, such as increasing welfare policies, preparing festival gifts, providing meal allowances, etc. Performance Evaluation mechanism should be established for employees who have long been working in the industry, such as carrying out quarterly or annual evaluations in care service institutions, and grant medals or bonuses to workers with outstanding performance, so as to enhance their sense of honor. For the students from elderly care-related vocational and technical colleges, we need to keep track on their views and visions of the industry, and conduct surveys regularly to fully understand the expectations of young potential practitioners in the elderly care industry and try the best to live up to such expectations. In this way, the next-generation of workers will fully believe in the future prospects of this career and choose to stay in this industry and contribute to the development of it.

On the other hand, there are certain limitations in this study. First of all, the data adopted by this research is the cross-sectional data of the survey on the care workers in Area B, Shanghai in 2019. Due to the Covid-19 lockdown, it is impossible to acquire the latest data via field research, resulting in data lag in reaching the conclusion. Secondly, there may be certain bias as the selected measurement questions are mainly based on the subjective evaluation of care workers themselves.

## Supplementary Information

**Additional file 1.**

## Data Availability

The datasets generated and/or analysed during the current study are not publicly available due the data used comes from a private database. but are available from the author on reasonable request.
