# Supplementary material for: Influencing factors of the treatment level of elderly care workers and their career development prospects
Source: BMC Geriatr. 2023 Jun 9;23:359. doi: 10.1186/s12877-023-04084-w (PMC10251313; doi:10.1186/s12877-023-04084-w)
Supplement: Supplementary file 1 — Additional file 1. [file 12877_2023_4084_MOESM1_ESM.docx]

***The construction of nursing staff for the elderly Questionnaire***

In order to further understand the current situation of the construction of nursing staff in Area B of Shanghai, improve the quality of institutional services and protect the rights and interests of nursing staff, the research team is now conducting a comprehensive study on the construction of nursing staff in Shanghai. The questionnaire is anonymous, and the information you provide will be used for scientific research only and will be kept completely confidential, so please fill out the questionnaire according to your actual situation and your heart's desire. Thank you for your support and have a nice life!

**Shanghai University of Engineering Science**

**1.Do you have any relevant work experience before coming to work in a senior living facility?【 】**

1）Yes 2）No

**2.How many years have you been working in the elderly service industry?【 】**

1）0-1years 2）1-2years 3）2-3years 4）3-5years 5）5-8years 6）8-10years 7）10 years or more

**3.What motivates you to work in this field? (Multiple choice allowed)【 】**

1）Awareness of respecting and serving the elderly 2）Family financial pressure 3）Relatively possessing skills in caring for the elderly 4）Other

**4.How many years have you worked in this organization?【 】**

1）0-1years 2）1-2years 3）2-3years 4）3-5years 5）5-8years 6）8-10years 7）10 years or more

**5.What is the main reason why you choose to work in this senior care facility? (Multiple choice is allowed)【 】**

1）Close to home 2）Convenient transportation 3）Parents staying here 4）Good environment 5） High salary and benefits 6）Distribution 7）Other

**6.Have you participated in nursing skills training?【 】**

1）Nursing care program training 2）Nursing care (medical care) training 3）No

**7.Do you have a professional qualification for nursing care?【 】**

1）Yes 2）No

**If yes, what is your certificate level?【 】**

1）Elementary (Grade 5) 2）Intermediate (Grade 4) 3）Advanced (Grade 3)

**8.Do you enjoy the relevant training subsidies? 【 】**

1）Yes, the proportion of subsidy is large 2）Yes, the proportion of subsidy is small 3）No

**9.Are you willing to participate in nursing skills training?【 】**

1）Very willing 2）More willing 3）Generally 4）Less willing 5）Not willing

**10.What do you think is the most lacking in your professional ability?【** 】

1）Theoretical learning 2）Nursing practice 3）Innovative service 4）Working motivation

**11.Do you have the qualification of long-term care insurance service?【 】**

1）Yes 2）No 3）Never heard of it

**12.Do you receive on-the-job training from this organization?【 】**

1）Yes, regularly 2）Yes, irregularly 3）No

**13.Have you participated in any relevant vocational skills competitions?【 】**

1）Yes,Name： 2）No

**14.What kind of work are you currently engaged in?【 】**

1）Nurse practitioner 2）Nursing caregiver (medical care) 3）Nursing caregiver 4）Other

**15.What kind of services do you provide for the elderly?【 】**

1）Institutional services 2）Community services 3）Home services 4）Other

**16.What services do you provide for the elderly? (Multiple choice)【 】**

1）Living care 2）Technical care 3）Rehab care 4）Psychological care 5）Training guidance 6）Care management 7）Other

**17.How long do you have to work every day?【 】**

1）3-5 hours 2）5-8 hours 3）8-10 hours 4）10 hours or more

**18.Are you required to work overtime frequently?【 】**

1）Often 2）Generally 3）Seldom 4）Basically not 5）Never

**19.If you work overtime, do you get overtime pay? 【 】**

1）Yes, regularly 2）Yes, irregularly 3）No 4）Other

**20.How many days of rest do you have in a month on average?【 】**

1）1 day 2）2days 3）3days 4）4days 5）5days 6）6days 7）Other

**21.How many elderly people do you take care of each day on average? 【 】**

1）5 people and below 2）6-10 people 3）11-15people 4）16people and above

**22.How do you feel about your treatment and salary level?【 】**

1）High, reasonable 2）Average, reasonable 3）Low, unreasonable 4）Especially low, very unreasonable 5）Other

**23.What kind of establishment do you belong to?【 】**

1）Career establishment 2）Labor contract 3）Other

**24.What are the social insurance items handled by the unit for you?(Multiple choice)【 】**

1）Pension insurance 2）Medical insurance 3）Unemployment insurance 4）Industrial injury insurance 5）Maternity insurance 6）Accidental injury insurance 7）Housing fund 8）Other

**25.What special protection and services do you receive from your employer?**

**_________________________________________________________________________**

**26.What difficulties do you have in your nursing work? (Multiple choice)【 】**

1. Low salary and long working hours 2）Difficulty in communicating with the elderly 3）Not respected by the society 4）Not respected by the elderly and their families 5）Poor welfare conditions 6）No improvement in salary for a long time 7）Difficulty in employment in other places 8）Other

**27.Do you have volunteers to serve in your daily work?【 】**

1）Often 2）Sometimes 3）Seldom 4）Basically not 5）Never

**28.Do you think the volunteers come to serve in the organization is very useful?【 】**

1）Good 2）Overall 3）Nothing 4）Low 5）I don't know

**29.How do you think the public support this elderly institution?【 】**

1）Very supportive 2）More supportive 3）Average 4）Not very supportive 5）Not supportive

**30.How do you think the public is satisfied with your service?【 】**

1）Very satisfied 2）Satisfied 3）Generally satisfied 4）Not very satisfied 5）Not satisfied

**31.How do you think your career development prospects are?【 】**

1）very optimistic, more room for upward mobility 2）general, not much transfer 3）more limited, more professional restrictions 4）very unpromising, salary and skills acquisition are problems 5）other

**32.What do you think are the factors that prevent you from working? (Multiple choice)【 】**

1）Low salary 2）High work intensity 3）High psychological pressure 4）High pressure on family care 5）High demand for care skills 6）Bad working atmosphere of colleagues 7）Bad environment of nursing home 8）Others

**33.If you have the opportunity, will you choose to jump from one job to another?【 】**

1）Will definitely 2）Will be willing, but will use discretion 3）Normally will not 4）Never will 5）No

**If there is a job-hopping, you will choose**【 】

1）Other industries 2）Other organizations of the same type 3）Other senior care service fields

**34.What do you suggest to this unit? (Multiple choice)【 】**

1）Strengthen the convenience of infrastructure 2）Improve the content of senior care services 3）Improve the daily management mechanism 4）Seek cooperation with public welfare organizations 5）Strengthen the admission standard of service personnel 6）Improve the salary and welfare of staff 7）Strengthen the skill training and assessment of service personnel 8）Improve the combination of medical and health care in the institution 9）Other

**Basic information**

**1.The name of the nursing home you are in:**

**2.Age：**

**3.Gender：【 】**

1）Male 2）Female

**4.Education level【 】**

1）Elementary school and below 2）Junior high school 3）High school 4）Secondary school or technical school 5）College 6）Bachelor's degree and above

**5.Your marital status：【 】**

1）Married 2）Widowed 3）Divorced 4）Unmarried

**6.How many children do you have now?【 】**

1）0 2）1 3）2 4）3and above

**7.What is your current living style?【 】**

1）Spouse living together 2）Spouse and children living togethe 3）Children living togethe 4）Living alone 5）Institute

**8.Your current income：【 】**

1）Less than 1000RMB 2）1000-2000RMB 3）2000-3000RMB 4）3000-4000RMB 5）4000-5000RMB 6）5000-6000RMB 7）6000-7000RMB 8）7000-8000RMB 9）8000RMB and more

**9.Your occupation before coming to this institution：【 】**

1）Agriculture, forestry, animal husbandry, fishery, water conservancy 2）Commercial, service industry personnel 3）State organs, party organizations, enterprises and institutions in charge 4）All kinds of professional and technical personnel 5）Officers and related personnel 6）Production, transportation equipment operators and related personnel 7）Military personnel 8）Other

**10.The nature of your account?【 】**

1）Shanghai urban hukou 2）Shanghai agricultural hukou 3） Provincial agricultural hukou 4） Provincial urban hukou

**11.The district and county where your household is located is: (only for Shanghai households)【 】**

1）Huangpu District 2）Xuhui District 3）Changning District 4）Jing'an District 5）Putuo District 6）Hongkou District 7）Yangpu District 8）Baoshan District 9）Minhang District 10）Jiading District 11）Pudong New District 12）Songjiang District 13）Jinshan District 14）Qingpu District 15）Fengxian District 16）Chongming District

**12.The district and county where you live now is：【 】**

1）Huangpu District 2）Xuhui District 3）Changning District 4）Jing'an District 5）Putuo District 6）Hongkou District 7）Yangpu District 8）Baoshan District 9）Minhang District 10）Jiading District 11）Pudong New District 12）Songjiang District 13）Jinshan District 14）Qingpu District 15）Fengxian District 16）Chongming District

**Thank you very much for your support!**
